# A Multiscale Evaluation of the Coupling Relationship between Urban Land and Carbon Emissions: A Case Study of Chongqing, China

**DOI:** 10.3390/ijerph17103416

**Published:** 2020-05-14

**Authors:** Chuanlong Li, Yuanqing Li, Kaifang Shi, Qingyuan Yang

**Affiliations:** 1 College of Resources and Environment, State Cultivation Base of Eco-agriculture for Southwest Mountainous Land, Southwest University, Chongqing 400715, China; lclabc1978@163.com; 2 Chongqing Jinfo Mountain Field Scientific Observation and Research Station for Kaster Ecosystem, Ministry of Education, Southwest University, Chongqing 400715, China; xslyq@foxmail.com; 3 Green Low Carbon Research Institute, School of Geographical Sciences, Southwest University, Chongqing 400715, China; 4 Chongqing Engineering Research Centre for Remote Sensing Big Data Application, School of Geographical Sciences, Southwest University, Chongqing 400715, China

**Keywords:** coupling relationship, urban land, carbon emissions, scale comparison, Chongqing

## Abstract

Exploring the coupling relationship between urban land and carbon emissions (CE) is one of the important premises for coordinating the urban development and the ecological environment. Due to the influence of the scale effect, a systematic evaluation of the CE at different scales will help to develop more reasonable strategies for low-carbon urban planning. However, corresponding studies are still lacking. Hence, two administrative scales (e.g., region and county) in Chongqing were selected as experimental objects to compare and analyze the CE at different scales using the spatiotemporal coupling and coupling coordination models. The results show that urban land and carbon emissions presented a significant growth trend in Chongqing at different scales from 2000 to 2015. The strength of the spatiotemporal coupling relationship between urban land and total carbon emissions gradually increased with increasing scale. At the regional scale, the high coupling coordination between urban land and total carbon emissions was mainly concentrated in the urban functional development region. Additionally, the high coupling coordination between urban land and carbon emission intensity (OI) was still located in the counties within the metropolitan region of Chongqing, but the low OI was mainly distributed in the counties in the northeastern and southeastern regions of Chongqing at the county level. This study illustrates the multiscale trend of CE and suggests differentiated urban land and carbon emission reduction policies for controlling urban land sprawl and reducing carbon emissions.

## 1. Introduction

Rapid urbanization has become an important factor affecting the carbon cycle and global climate change [1,2,3,4,5,6]. The International Energy Agency reported that urban areas contributed approximately 71% of global carbon emissions [7], which has attracted the attention of all countries in the world [8,9,10,11]. As the largest carbon emitter in the world, China proposed developing low-carbon urbanization to achieve the coordination of urbanization and carbon emissions [12,13,14]. Built-up area (hereafter referred to as urban land), as the carrier of land urbanization, represents the external representation of the impact of human activities on carbon emissions [8]. Although many studies have indicated that the correlation between urbanization and carbon emissions is complex and diverse, one of the most popular objects of analysis is the land urbanization–carbon emission relationship [15,16]. Currently, since inefficient land urbanization in China has resulted in disorderly urban sprawls [9,17,18,19], leading to more carbon emissions, how to coordinate the relationship between urban land and carbon emissions is a major problem to be solved that is of great significance for sustainable development in China.

In recent decades, a body of studies has addressed the relationship between urban land and carbon emissions from different perspectives [20,21]. The findings of these studies have shown that land urbanization involves changes in economic production, lifestyles, and land use, which affect carbon emissions [22]. In addition, the results also presented the different strengths of impacts of land urbanization on carbon emissions at various development stages, regions, and scales [23,24,25]. However, few studies have attempted to evaluate the effect of carbon emissions on land urbanization. Some studies have suggested that the effect of spatial agglomeration and human capital are two important transmission channels for environmental performance that affect the quality of urban development in China [26]. Therefore, the coupling relationship between urban land and carbon emissions has become an important issue in the process of urbanization [27,28]. Effectively quantifying and exploring the coupling relationship between urban land and carbon emissions will not only help to promote the coordinated development of urban land and carbon emissions but also reveal the impact mechanism of low-carbon urban development.

A few studies have evaluated the coupling relationship between urban land and carbon emissions. However, due to the availability of carbon emission data, corresponding results have been found at national, regional, or prefectural scales [25], but not at smaller scales, such as the county scale. As the county is the basic unit of economic and social development in China [29], evaluation of the coupling relationship at the county scale is conducive to the adjustment of regional urban land use structure and layout optimization and promotes low-carbon development of the regional economy and society. Additionally, the coupling relationship also shows the spatiotemporal changes across scales. Many studies have suggested that the same theory should not be applied to different scales, especially at different administrative scales [30,31]. The urban land–carbon emission coupling relationship at different scales is still unknown in China.

To address the above deficiencies, Chongqing, which is only municipality in the western region of China and has experienced rapid urban expansion and carbon emission growth [32], was selected as the study area. County socioeconomic development in Chongqing presents obvious spatial differences, and regional differences can be regarded as a microcosm of China [33]. Thus, exploring the coupling urban land–carbon emission relationship could provide a significant reference for, and contribution to, other areas in China. In summary, the contributions made by this study are as follows. First, the coupling relationship between urban land and carbon emissions was first evaluated at the county scale. Second, a scale comparison of the coupling relationship was revealed from different perspectives. To achieve these objectives, first, data collection and processing were conducted. Then, spatiotemporal coupling and coupling coordination models were calculated to quantify the coupling relationship between urban land and carbon emissions. This study provides a new framework and methodology to develop a sustainable development model for low-carbon cities.

## 2. Study Area and Data Sources

### 2.1. Study Area

**Figure 1:**
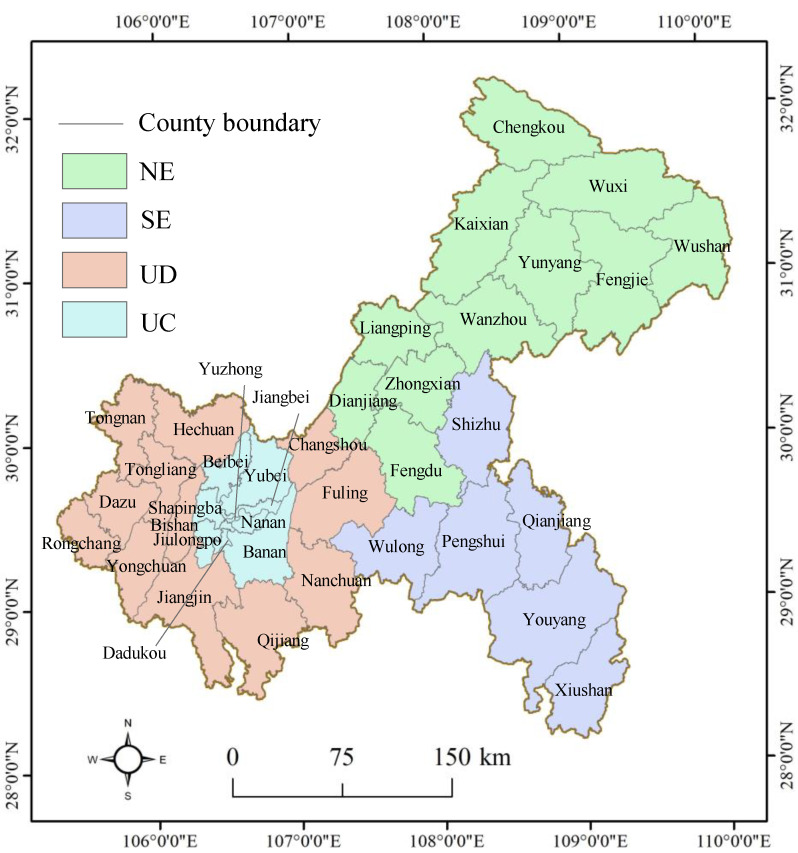
Location of the study area. Note: NE represents the northeastern region of Chongqing; SE represents the southeast region of Chongqing; UD represents the urban functional development region; UC represents the metropolitan region of Chongqing.

Chongqing is located between 105°17’–110°11’ E and 28°10’–32°13’ N, covering an area of 82,400 km^2^. The jurisdiction of Chongqing is 450 km wide from south to north and 470 km long from east to west; Chongqing is divided into 38 counties (or districts) (Figure 1). As Chongqing is the largest municipality with the largest area and the largest population in China, it includes large cities, large rural areas, large reservoirs, large mountainous areas, and various ethnic regions. Considering its population, resources, environment, economy, society, culture, and other factors, Chongqing is divided into five functional regions: the northeastern region of Chongqing (NE), the southeast region of Chongqing (SE), the urban functional development region (UD), the metropolitan core region, and the metropolitan development region. Note that the metropolitan core region and the metropolitan development region are regarded as the metropolitan region of Chongqing (UC) when considering the nesting function between administrative units. Due to its rapid socioeconomic development, environmental effects, especially carbon emissions, are becoming increasingly serious in the process of urbanization, and Chongqing has been chosen as one of the low-carbon pilot cities in China. Therefore, it is urgent to determine whether there is coordinated development between urban land and carbon emissions in Chongqing. To answer this question, two representative scales (e.g., regions and counties) were selected as experimental objects to evaluate the coupling relationship between urban land and carbon emissions.

### 2.2. Data Sources

**Figure 2:**
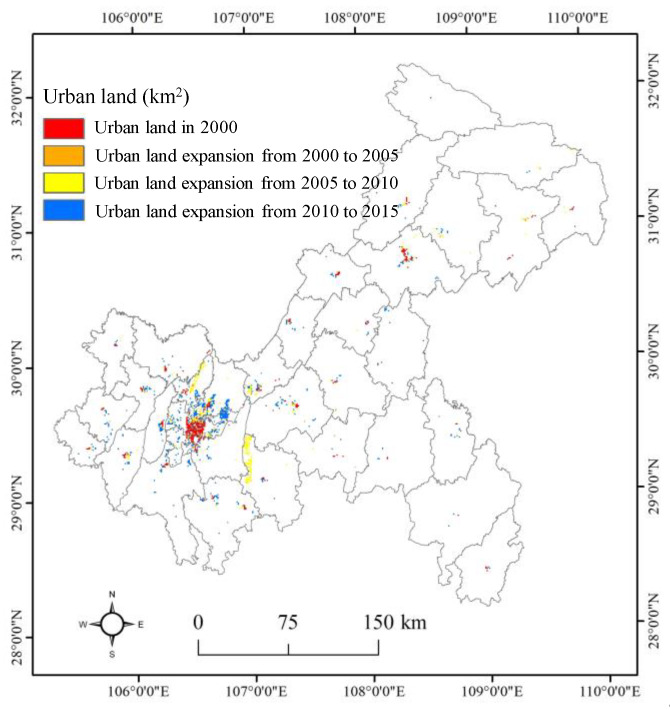
Urban land expansion in Chongqing from 2000 to 2015.

**Figure 3:**
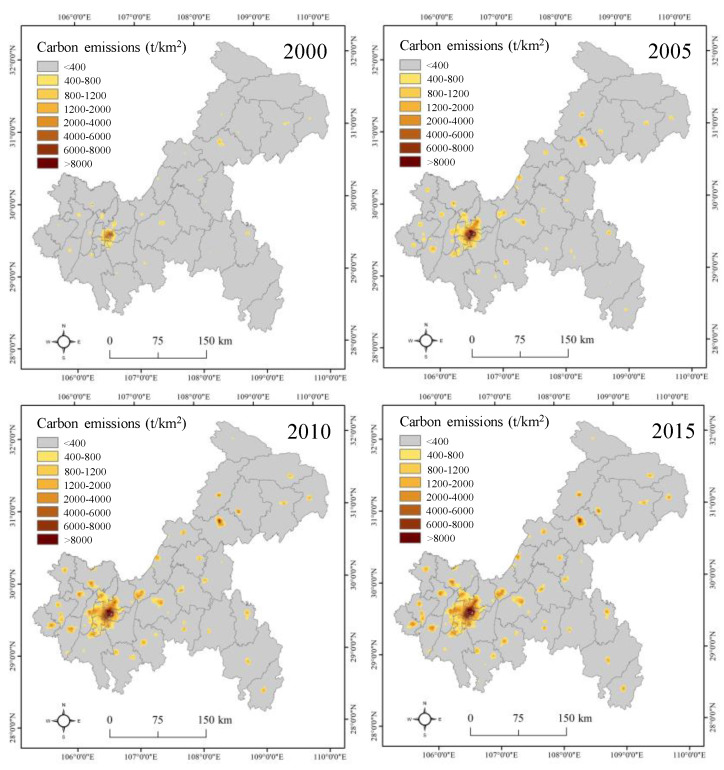
Carbon emissions in Chongqing from 2000 to 2015.

Urban land, carbon emission data, and administrative division vectors were collected and preprocessed in this study. Urban land data were extracted from the land use/cover datasets obtained from the Data Centre for Resources and Environmental Sciences, Chinese Academy of Sciences (http://www.resdc.cn). Note that urban built-up areas and other built-up areas were merged as urban land because these land-use types are closely related to carbon emissions (Figure 2). Then, carbon emission data were collected from the Open-source Data Inventory (ODIAC) [34]. The data were estimated from the combination of multisource remote sensing data, nonpoint source data, statistical carbon emission data from fossil fuel consumption (Figure 3), and passed an accuracy verification [35]. The ODIAC carbon emission data have been widely used in the carbon cycle and low-carbon cities in the international academic community [36,37]. The administrative division vector of Chongqing was obtained from the National Geomatics Centre of China, and then regional boundaries were merged from county data in Chongqing. Finally, all the data were projected into an Albers equal-area conic projection with reference to the WGS84 datum, and the spatial data were resampled to a spatial resolution of 1 km.

## 3. Study Area and Data Sources

### 3.1. Spatiotemporal Coupling Model

The spatiotemporal coupling model was used to evaluate the degree of interaction between urban land and carbon emissions. Since the geometric centers between urban land and carbon emissions in Chongqing were separated spatially, the spatiotemporal coupling model was defined as spatial overlaps between different geometric centers [25]. The specific formulas are as follows:

(1) $$X_{U\;}=\frac{\sum_{i=1}^{n}U_{i}x_{i}}{\sum_{i=1}^{n}U_{i}}$$

(2) $$Y_{U}=\frac{\sum_{i=1}^{n}U_{i}y_{i}}{\sum_{i=1}^{n}U_{i}}$$

(3) $$X_{C}=\frac{\sum_{i=1}^{n}C_{i}x_{i}}{\sum_{i=1}^{n}C_{i}}$$

(4) $$Y_{C}=\frac{\sum_{i=1}^{n}C_{i}y_{i}}{\sum_{i=1}^{n}C_{i}}$$

(5) $$T=\sqrt{{{(X}_{U}{-X}_{C})}^{2}{+(Y}_{U}{-Y}_{C}{)}^{2}}$$

where *X_U_* and *Y_U_* represent the longitude and latitude, respectively, of the geometric centers in urban land; *X_C_* and *Y_C_* represent the longitude and latitude, respectively, of geometric centers in carbon emissions; *U_i_* and *C_i_* represent urban land and carbon emissions in *i* county or region, respectively; *x* and *y* represent the longitude and latitude, respectively; *n* is the number of subjects; and *T* represents the spatiotemporal coupling degree. The shorter the distance is, the higher the overlap and the stronger the coupling.

### 3.2. Coupling Coordination Model

The coupling coordination model was employed to quantify the degree of coupling coordination between urban land and carbon emissions in the development process. Because different systems are affected by both external and internal impacts, the degree of coupling coordination represents the degree of harmony between the urban land system and the carbon emission system. According to Li et al. [38] and Li et al. [25], urban land and carbon emissions represent the level of the urban land system and the carbon emission system, respectively. Note that before the calculation, the indexes need to be normalized:

(6) $$B=\frac{{N-N}_{\min}}{N_{\max}{-N}_{\min}}$$

where *B* is the normalized value; *N* represents the value of urban land or carbon emissions; and *N_min_* and *N_max_* represent the minimum and maximum values of indicators, respectively.

(7) $${X=(O\times Z)}^{1/2}$$

(8) $$O=\frac{2\sqrt{UC}}{U+C}$$

(9) Z=αU+βC

where *X* represents the coupling coordination degree and *α* and *β* represent regression parameters. Based on Li et al. [38], both parameters were set to 0.5.

## 4. Results and Discussion

### 4.1. Spatiotemporal Characteristics of Urban Land and Carbon Emissions

**Figure 4:**
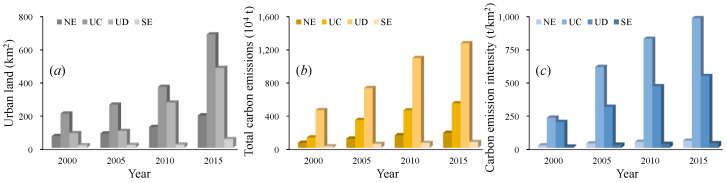
Urban land and carbon emissions at the regional scale in Chongqing from 2000 to 2015. Note: (**a**) urban land; (**b**) total carbon emissions; (**c**) carbon emission intensity.

**Figure 5:**
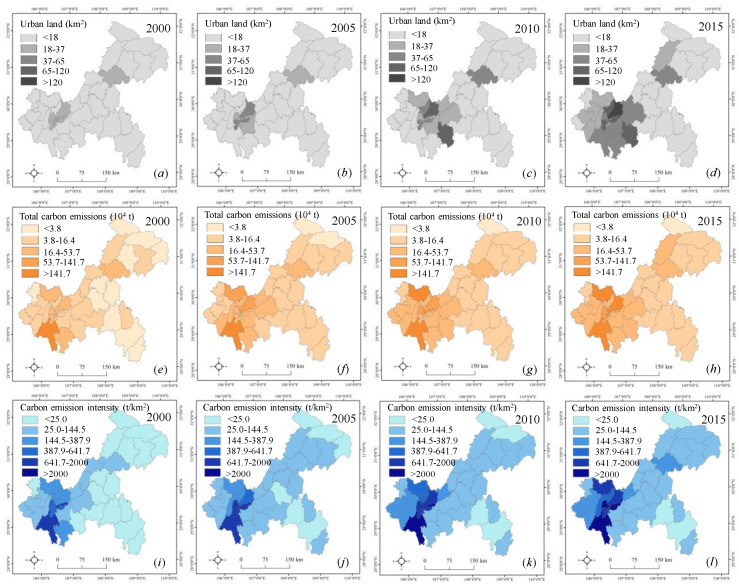
Spatiotemporal variations of urban land and carbon emissions at the county scale in Chongqing from 2000 to 2015. Note: (**a**)–(**d**) urban land; (**e**)–(**h**) total carbon emissions; (**i**)–(**l**) carbon emission intensity.

As shown in Figure 4 and Figure 5, we found that urban land presented a significant growth trend in Chongqing from 2000 to 2015 at the county and regional scales. At the regional scale, urban land expansion was concentrated in the UD and UC, with values from 88 km^2^ and 206 km^2^ in 2000 to 272 km^2^ and 367 km^2^ in 2015, respectively. In contrast, less urban land expansion occurred in the SE and NE from 2000 to 2015. At the county level, we found that urban land was generally dominant in Yuzhong, Yubei, Jiangbei, Jiulongpo, and Wanzhou but was less distributed in other counties from 2000 to 2005. Then, urban land presented a rapid growth trend in the counties within the UD and UC from 2010 to 2015, but there was still a slow growth trend in other counties. On the whole, the evolution pattern of urban land expansion presented a similar trend at the county and regional scales, but the smaller the scale was, the more heterogeneous the regional urban land pattern. The reasons for the above phenomena are as follows. Because the population and businesses are mainly concentrated in the counties within the UC and UD, housing demand could partly stimulate urban land expansion. Additionally, rapid social and economic development has promoted the construction of development zones, leading to more urban land expansion.

Figure 4 and Figure 5 indicate a slight difference in total carbon emissions and carbon emission intensity in Chongqing at different scales from 2010 to 2015. At the regional scale, total carbon emissions in the UD rapidly increased from 453 × 10^4^ t in 2000 to 1260 × 10^4^ t in 2015, and then carbon emission intensity gradually increased from 194 t/km^2^ in 2000 to 540 t/km^2^ in 2015. In addition, we found that total carbon emissions in the UC gradually increased from 124 × 10^4^ t in 2000 to 536 × 10^4^ t in 2015, and then carbon emission intensity sharply increased from 227 t/km^2^ in 2000 to 976 t/km^2^ in 2015. At the county level, total carbon emissions were concentrated in Jiangjin and Hechuan within the UD from 2000 to 2015, while the counties with high carbon emission intensity were still mainly located in Yuzhong, Jiangbei, Yubei, Dadouk, and Jiulongpo within the UC. The reason may be that with the gradual transfer of Chongqing’s industrial layout to the UD from the UC, the counties represented by Jiangjin have undertaken the industrial transfer of the UC, resulting in more carbon emissions. The counties within the UC still represent the social-economic center of Chongqing, attracting increasingly more people and enterprises, which would lead to a high level of carbon emission intensity [39].

### 4.2. Spatiotemporal Coupling Relationship between Urban Land and Carbon Emissions

As listed in Table 1, the shift distance between urban land and total carbon emissions (UL–TC) and the shift distance between urban land and carbon emission intensity (UL–CI) gradually decreased over time. The spatiotemporal coupling relationship between urban land and carbon emissions (SC) gradually increased. Specifically, the UL–TC decreased from 33.96 km in 2000 to 18.40 km in 2015, and the UL–CI decreased from 56.50 km in 2000 to 41.52 km in 2015 at the regional scale. The UL–TC decreased from 53.76 km in 2000 to 36.30 km in 2015, and the UL–CI decreased from 52.86 km in 2000 to 40.72 km in 2015 at the county scale. We found that the spatiotemporal coupling degree of urban land and total carbon emissions (ST) was higher than that of urban land and carbon emissions intensity (SI). Generally, the strength of the ST gradually increased with increasing scale. This is because the smaller the scale is, the stronger the spatial heterogeneity, resulting in a larger gap between urban land and carbon emissions.

**Figure 6:**
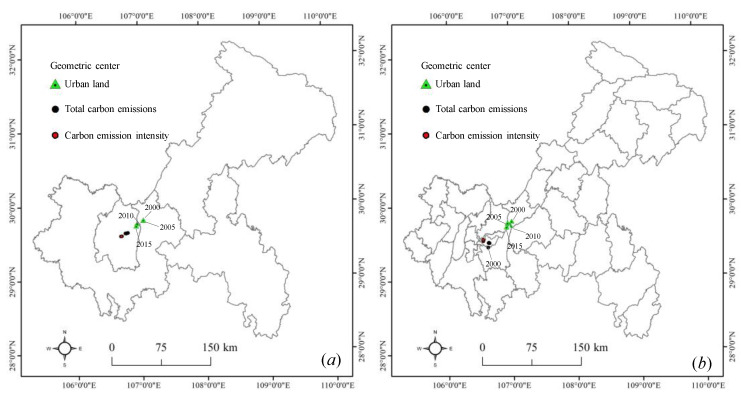
The geometric center shift of urban land and carbon emissions in Chongqing. Note: (**a**) regional scale; (**b**) county scale.

From Figure 6a, the geometric center of urban land was moving toward the southwest at the regional scale. This may be because urban expansion was mainly concentrated in the UC. The geometric centers of total carbon emissions and carbon emissions intensity remain stable. From the spatiotemporal perspective, the ST was higher than that of SI, which was consistent with the results in Table 1. From Figure 6b, we found that the geometric center of urban land moved slowly to the southwest at the county scale, and the geometric centers of carbon emissions kept relatively stable positions within Yuzhong and Nanan. It has been demonstrated that due to socioeconomic development, urban land presented a large-scale expansion in these counties. Since the population and industrial enterprises are still concentrated in the counties within the UC and UD, the spatial pattern of carbon emissions has not significantly changed.

### 4.3. Coupling Coordination between Urban Land and Carbon Emissions

**Figure 7:**
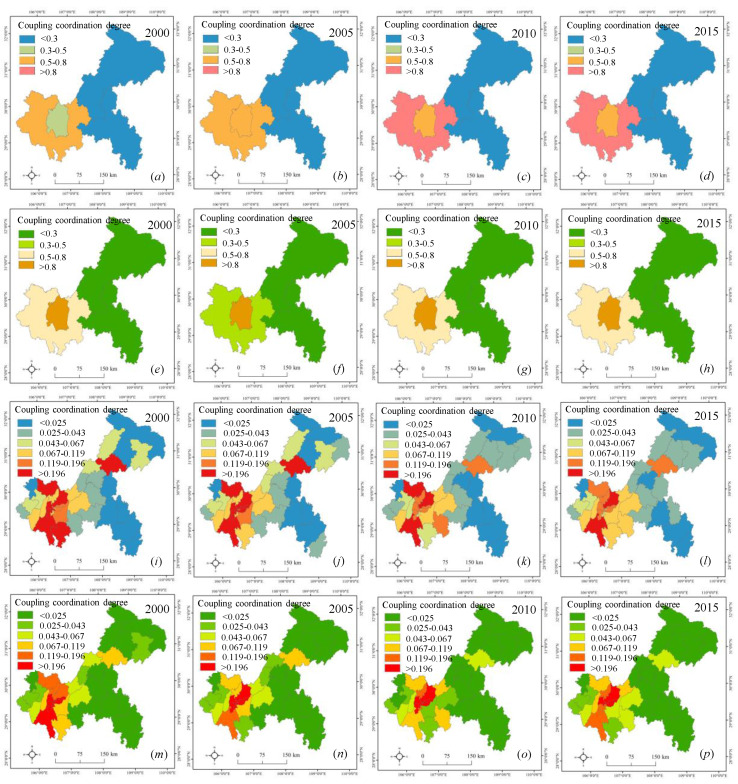
The coupling coordination degree between urban land and carbon emissions at different scales. Note: (**a**)–(**d**) the coupling coordination degree between urban land and total carbon emissions at the regional scale; (**e**)–(**h**) the coupling coordination degree between urban land and carbon emission intensity at the regional scale; (**i**)–(**l**) the coupling coordination degree between urban land and total carbon emissions at the county scale; (**m**)–(**p**) the coupling coordination degree between urban land and carbon emission intensity at the county scale.

The coupling coordination between urban land and carbon emissions (CC) at diffident scales is shown in Figure 7. The spatial distribution of CC was consistent with that of urban land and carbon emissions. The high value areas of carbon emissions and urban land expansion were also the areas with the high value of CC. On the contrary, the areas with a low value of CC were also the areas with low carbon emissions and urban land. This was because their industrial structure remained to be optimized, and their carbon emissions and urban land increased relatively slowly. As illustrated in Figure 7a–d, from 2000 to 2015, the high coupling coordination between urban land and total carbon emissions (OT) was mainly concentrated in the UD, followed by the UC. The UD is the main battlefield of Chongqing’s future economic development. On the one hand, the region bore the industrial transfer from the UD, leading to high carbon emissions. On the other hand, the rapid economic development and population growth have also spread to the UD, resulting in rapid urban expansion. The low OT was mainly located in the NE and SE. The reason for this was that the NE and SE are the ecological protection regions, which should adhere to the idea of “point development”, resulting in low carbon emissions and urban expansion. Also, we found the highest coupling coordination between urban land and carbon emission intensity (OI) was mainly concentrated in the UC (Figure 7e–h). This implied that the UC is still the social and economic center of Chongqing, with the highest carbon emission density.

The OT at the county scale is presented in Figure 7i–l. The high value was mainly concentrated in Jiangjin, Hechuan, Yubei, and Wanzhou, which were hot counties with high social and economic development. Many energy consuming enterprises are distributed in these counties, which would result in lots of carbon emissions [39]. Additionally, the high OI was still located in the counties within the UC, but the low OI was mainly distributed in the counties within the NE and SE (Figure 7m–p). By and large, the CC presented a decrease trend as time goes on. This meant that an obvious dislocation between urban expansion and carbon emission in Chongqing at the county scale.

From Figure 7, we found that although the CC has changed with the change of scales, the spatiotemporal patterns kept relatively stable. This indicated that the dual spatial structure (high–low structure) of CC has not changed in Chongqing from 2000 to 2015. There were obvious differences for OT and OI at county and regional scales due to the effects of scale dependence and scale effect [40]. By comparing and analyzing the CC from different scales, it could not only provide a basis for Chongqing to formulate low carbon urban planning system from the macro level, but also provide a scientific reference for local governments to formulate differentiated urban planning and emission reduction policies at the county scale.

### 4.4. Limitations and Future Directions

A few limitations should be mentioned and addressed in the future. First, the driving mechanism has not been discussed and compared within different scales. Some studies have indicated that the urbanization rate has become an important factor in the coupling relationship between urban land and carbon emissions. Rapid urbanization may lead to a decline in coupling coordination between urban land expansion and carbon emissions. When the urbanization rate reaches a certain level, the management and technology level is relatively high, and improvement in the urbanization level can effectively inhibit the increase in carbon emissions. The situation of economic growth driven by land has fundamentally changed, increasing coupling. Thus, the industrial structure, population density, and gross domestic product will also be considered in future studies exploring the driving mechanism. Second, only Chongqing was selected as a case study to quantify the coupling relationship at different scales. In the future, we will attempt to analyze the relationship between urban land and carbon emissions in China across regional, provincial, prefectural, and county scales. Third, coarse-resolution (1 km) urban land data derived from land use/cover data could not be used to identify the details of the city. Remote sensing data with a high spatial resolution, including Landsat Thematic Mapper (TM)/Enhanced Thematic Mapper Plus (ETM+), Quick Bird, and Systeme Probatoire d’Observation de la Terre (SPOT) images, will be employed to accurately extract urban land in future studies. Finally, carbon sources from other objects and carbon sinks will also be adopted to evaluate the urban land–carbon emission relationship.

## 5. Conclusions

This study evaluated the coupling relationship between urban land and carbon emissions in Chongqing at regional and county scales from 2000 to 2015 and explored the scale effect of urban land and carbon emissions, which provided a profound understanding of the balance between urban development and environmental effects. First, urban land and carbon emissions were extracted from land use/cover datasets and ODIAC data. Then, spatiotemporal coupling and coupling coordination models were employed to quantify the coupling relationship. The results show that urban land and carbon emissions presented a significant growth trend in Chongqing from 2000 to 2015 at the county and regional scales. The ST was higher than the SI, and the strength of the ST gradually increased with increasing scale. The spatial distribution of CC was consistent with that of urban land and carbon emissions. At the regional scale, the high OT was mainly concentrated in the UD and then in the UC. The high OI was still located in the counties within the UC, but the low OI was mainly distributed in the counties within the NE and SE.

Our findings have several implications for policymakers to control urban land sprawl and reduce carbon emissions. For areas with large urban land areas and high carbon emissions, strict land use controls should be adopted to delineate reasonable urban development boundaries. Different urban land and carbon emission reduction policies should be formulated and implemented according to the different stages of urbanization. Finally, we should consider the coupling relationship at different scales from a global perspective and consider the spatial spillover effect of carbon emissions.

## Figures and Tables

**Table 1:** The shift distance between urban land and carbon emissions at different scales.

Scale	Variable	Distance (km^2^)			
		2000	2005	2010	2015
Regional scale	UL–TC	33.96	30.68	22.56	18.40
	UL–CI	56.50	55.49	45.08	41.52
County scale	UL–TC	53.76	41.10	43.16	36.30
	UL–CI	52.86	45.16	47.53	40.72

Note: UL–TC represents the shift distance between urban land and total carbon emissions; UL–CI represents the shift distance between urban land and carbon emission intensity.

## Abbreviations

**Full name**	**Abbreviation**
Coupling relationship between urban land and carbon emissions	CE
The northeastern region of Chongqing	NE
The southeast region of Chongqing	SE
The urban functional development region	UD
The metropolitan region of Chongqing	UC
The shift distance between urban land and total carbon emissions	UL–TC
The shift distance between urban land and carbon emission intensity	UL–CI
Spatiotemporal coupling relationship between urban land and carbon emissions	SC
Spatiotemporal coupling degree of urban land and total carbon emissions	ST
Spatiotemporal coupling degree of urban land and carbon emissions intensity	SI
Coupling coordination between urban land and carbon emissions	CC
Coupling coordination between urban land and total carbon emissions	OT
Coupling coordination between urban land and carbon emission intensity	OI
